# The Mediating Effect of Physical Function Decline on the Association Between Social Activity and Cognitive Function in Middle and Older Korean Adults: Analyzing Ten Years of Data Through Multivariate Latent Growth Modeling

**DOI:** 10.3389/fpsyg.2020.02008

**Published:** 2020-12-18

**Authors:** Sung Man Bae

**Affiliations:** Department of Psychology and Psychotherapy, Dankook University, Cheonan, South Korea

**Keywords:** social activity, physical function, cognitive function, latent growth modeling, elderly

## Abstract

**Purpose:**

This study aimed to examine the long-term association between social activity, physical function decline and cognitive function, as well as verify the long-term mediating effect of physical function decline on the relationship between social activity and cognitive function.

**Methods:**

Data from the Korean Longitudinal Study of Aging (KLoSA) that was collected over 10 years was analyzed. The sample included 10,240 adults aged 45–93 years (Mean age = 61.66 [SD = 11.061]). Multivariate latent growth modeling (LGM) was applied to verify the long-term effect of social activity and physical function on cognitive function.

**Results:**

The results revealed that social activity had a positive impact on cognitive function and negative impact on physical function decline after controlling for age and education level. Additionally, physical function decline negatively influenced cognitive function. Finally, social activity indirectly affected cognitive function through physical function decline.

**Conclusion:**

The contribution of this study was to test the long-term effect social activity on physical and cognitive function.

## Introduction

Decreased physical and cognitive function are major issues associated with aging (Clouston et al., 2013; Kim, 2016). Therefore, researchers have explored modifiable variables that prevent physical and cognitive decline. Previous research has examined the relationship of social activity with physical and cognitive function (Glei et al., 2005; Bidzan et al., 2016; Frith and Loprinzi, 2017; Bidzan-Bluma and Lipowska, 2018; Dugan et al., 2018) and social activity may be a key factor mitigating physical and cognitive decline.

Social activity refers to various activities in social situations, such as participation in social organizations (e.g., civic organization), interactions with friends, and leisure and hobby activities (Glei et al., 2005; Bidzan et al., 2016; Su et al., 2018). Past studies have verified the effect of social activity on cognitive function and impairment (Hsu, 2007; Fu et al., 2018). In particular, the 3-year Monongahela-Youghiogheny Healthy Aging Team (MYHAT) study revealed that the frequency of engagement in social activities was negatively related to the risk of cognitive impairment (Hughes et al., 2013). In addition, a longitudinal and population-based study by Glei et al. (2005) revealed that social activities such as socializing with friends, performing volunteer work, and participating in religious, political, and elderly organizations helps preserve cognitive function in older adults.

Social activity could also mitigate decline in physical function. Social activities provide opportunities for maintaining physical function, and, as a result, they help preserve physical function (Tomioka et al., 2017). In other words, social activities may prevent motor function decline and loss of the ability to perform activities of daily living (Tomioka et al., 2016). Indeed, previous research has found that social activities (or social participation) are negatively associated with physical function decline (James et al., 2011; Fujihara et al., 2018; Tomioka et al., 2018).

Physical function is well known as a key predictor of cognitive function. Physical function is basic physical ability for activity and includes basic activities of daily living and instrumental activities of daily living (IADL) such as shopping, handling money, and transportation utilization (Bidzan et al., 2016; Bae et al., 2019). Actually, past studies verified the relevance between physical function and cognitive function (Grande et al., 2014; Vancampfort et al., 2017, 2018). Inversely, decreased physical function was positively related to cognitive impairment.

Based on previous research, social activity can directly influence cognitive function and may have an indirect impact on cognitive function through physical function (Glei et al., 2005; Fujihara et al., 2018; Su et al., 2018). However, there is a lack of understanding of the specific relationship between the three variables. In particular, the long-term mediating effect of physical function on the relationship between social activity and cognitive function has not been confirmed.

In order to accurately test the association between social activity, physical function, and cognitive function, confounding variables should be controlled. In particular, age and education are related to cognitive function. Previous studies have verified that age is positively related to cognitive impairment and education are negatively associated with cognitive decline (Grande et al., 2014; Xiang and An, 2015; Chen and Chang, 2016).

The object of this study was to verify the mechanism whereby social activity affects cognitive function. In other words, this study examined the long-term mediating effect of physical function decline on the relationship between social activity and cognitive function using multivariate latent growth modeling (LGM). LGM is a powerful method for analyzing the relationship between changes in latent factors over time.

## Materials and Methods

### Participants and Survey

Data from the Korean Longitudinal Study of Aging (KLoSA) (Shin et al., 2016) conducted by the Korea Employment Information Service, were used in this study. The KLoSA was performed biennially from 2006 to 2016 and included adults nationwide aged 45 and older. The KLoSA measured the social, economic, psychological demographic characteristics and health status of the elderly. The sample was extracted from 1,000 survey sites based on population proportions from the 2005 Population and Housing Census. Trained professional interviewers visited the household and explained the purpose of the survey. The survey was conducted using a computer assisted personal interviewing (CAPI) using a notebook computer. Participants signed consent forms and the interviewer confirmed that the subject completed the survey. Data from adults aged 45 to 93 were utilized in final analysis. A total of 10,240 people (Mean age = 61.66 years [SD = 11.06]; 4463 men, 5777 women) participated in 2006, and the response rate was 84.78% (Mean age = 63.62 years [SD = 10.88]; 8681 people; 3766 male, 4915 female) in 2008, 77.29% (Mean age = 65.25 years [SD = 10.52]; 7915 people; 3411 male, 4504 female) in 2010, 73.08% (Mean age = 66.79 years [SD = 10.20]; 7484 people; 3215 male, 4269 female) in 2012, 68.63% (Mean age = 68.29 years [SD = 9.90]; 7028 people; 2987 male, 4041 female) in 2014, and 64.62% (Mean age = 70.80 years [SD = 9.60]; 6617 people; 2781 male, 3836 female) in 2016. Reasons for dropouts included death, disease, and loss of contact, and the number of deaths in each wave were as follows: 187 people in the 2nd wave, 309 in the 3rd, 327 in the 4th, 438 in the 5th, and 403 in the 6th.

### Measures

#### Physical Function Decline

The Korean IADL was used to measure decline in physical function(Shin et al., 2016); this list is a revision of the original IADL by Lawton and Brody (1969). The inventory consists of ten items on a 3-point scale, ranging from 1 (can do without help) to 3 (need full help). The items address housework, cooking, shopping, washing clothes, utilization of public transportation, and handling money. Higher total scores indicate greater physical function decline.

#### Social Activity

The five items was used to measure the frequency of participation in social activities. This measure consists of seven items on a 10-point scale (1 = no activity, 6 = once a month, 10 = almost every day). The questionnaire assesses social activities including religious activity, volunteer work, political/civic organization activities, meeting with friends and acquaintances, and leisure/culture/sports organization activities (Lin, 2017). Higher total scores indicate more frequent participation in social activities.

#### Cognitive Function

In order to measure the degree of cognitive impairment, the Korean version of the Mini-Mental Status Exam was used (Kang et al., 1997). The measure consists of the following subscales: orientation, verbal memory, concentration and calculation, language, praxis, and visuospatial construction. Total scores range from 0 to 30, and a higher total score indicates higher cognitive function.

#### Control Variables

Age was measured as continuous variable ranging from 45 to 93 years. Education was coded as a nominal variable (1 = below middle school graduation, 2 = high school graduation or above). Sex was coded as a dummy variable (male = 0, female = 1).

### Analysis

**FIGURE 1:**
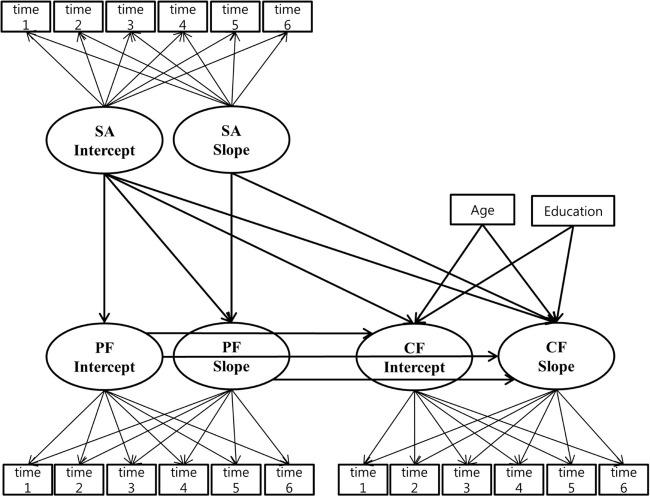
Multivariate latent growth model. PF, physical dysfunction; SA, social activity; CF, cognitive function.

Independent-sample *t*-tests and ANOVA were conducted to test the differences in cognitive function by age, gender, education, marital status, and religion in baseline (2006 year). LGM by the AMOS 20.0 program was used to verify the trajectories (change trend) in a variable and the associations between changes in the parameters of variables. Parameters comprise an intercept and slope. LGM can verify the direct effect of intercept of one latent factor on the intercept and slope of another latent factor. LGM also can test the direct effect of slope of one latent factor on the slope of another latent factor. In the first step, univariate LGM was performed to test the change trajectories in social activity, physical function decline, and cognitive function, and compared the fit of a no growth and a linear growth model. In the second step, multivariate LGM was conducted to test the relevance between latent factors. Full-information maximum likelihood, which is an efficient and unbiased method, was used to estimate parameters. The fit of the research model (Figure 1) was verified based on the chi-square value, the Tucker-Lewis index (TLI), comparative fit index (CFI), and root mean square error of approximation indices (RMSEA). TLI and CFI are deemed acceptable (good or excellent) if they have values higher than 0.95, and RMSEA is acceptable (good or excellent) if it is lower than 0.05 (Hong, 2000). Finally, A Bootstrapping test was conducted to examine the indirect effects of physical function decline on the relationship between social activity and cognitive function. The direct effect is significant when a zero value is not included in the confidence interval.

## Results

### *t*-Tests and ANOVA

Descriptive statistics such as minimum and maximum values, mean, and standard deviation are presented in Table 1. *T*-test results indicated that that cognitive function was higher in men than in women (*p* < 0.0001), higher in married people than those living alone (*p* < 0.0001), and higher in people with higher education than high school graduates (*p* < 0.0001), while people with religion had higher cognitive function than those without (*p* < 0.0001). As a result of ANOVA, there was no difference in cognitive function according to age (Table 2).

**TABLE 1:** Descriptive statistics of the variables.

	**Min**	**Max**	**Mean**	**SD**	***N***
PF1(2006)	10	50	11.63	5.801	10240
PF2(2008)	10	50	11.66	5.979	8681
PF3(2010)	10	50	11.84	6.602	7915
PF4(2012)	10	50	11.79	6.448	7483
PF5(2014)	10	50	11.92	6.671	7028
PF6(2016)	10	50	11.99	6.654	6617
SA1(2006)	0	32	4.30	4.463	10240
SA2(2008)	0	27	4.22	4.018	8681
SA3(2010)	0	28	3.82	3.748	7915
SA4(2012)	0	37	3.87	3.750	7483
SA5(2014)	0	31	3.96	3.646	7028
SA6(2016)	0	24	3.80	3.671	6617
CF1(2006)	0	30	25.43	5.324	10033
CF2(2008)	0	30	25.20	5.303	8370
CF3(2010)	0	30	25.12	5.514	7484
CF4(2012)	0	30	25.30	5.516	7112
CF5(2014)	0	30	25.09	5.605	6657
CF6(2016)	0	30	25.09	5.521	6278

*PF, Physical dysfunction; SA, Social activity; CF, Cognitive function.*

**TABLE 2:** *T* tests and ANOVA in MMSE for socio-demographic variables.

			**MMSE**		***p*-value**
	***N***	**%**	**Mean**	**SD**	
**Age**					<0.0001
45–54	3249	32.1	28.03	2.61	
55–64	2748	27.2	26.56	3.85	
65–74	2646	26.2	24.21	5.16	
≥75	1398	14.4	19.41	7.24	
**Gender**					<0.0001
Male	4463	43.5	26.64	4.36	
Female	5791	56.5	24.48	5.82	
**Education**					<0.0001
≤Elementary	4832	47.1	22.77	6.12	
Middle school	1657	16.2	27.06	3.29	
High school	2708	26.4	27.91	2.96	
≥College	1057	10.3	28.49	2.37	
**Marital status**					<0.0001
Married	7971	77.7	26.34	4.43	
Single (including separated, divorced)	2283	22.3	21.97	6.83	
**Religion**					
Yes	5680	55.4	25.71	4.97	<0.0001
No	4574	44.6	25.06	5.75	
Total	10254	100.0	25.42	5.34	
					

### The Changing Patterns of the Variables

A univariate LGM was performed to identify the trajectories of the variables, and the results are described in Table 3. Based on past studies and the mean trend in each variable, we compared the linear growth model and no growth model. First, the fitness of linear growth model of physical function decline was better than no growth model, which means that physical function decline increased linearly over time. Second, the fit of linear growth model of social activity was better than no growth model. In other words, social activity decreased linearly from wave 1 to 6. Third, the fitness of linear growth model of cognitive function was better than no growth model. That is, cognitive function decreased linearly over time.

**TABLE 3:** Comparisons of fitted growth curve models for the variables.

**Variable**	**Model**	**χ^2^ (df)**	**df**	**TLI**	**CFI**	**RMSEA**
Physical dysfunction	No growth	1764.505	12	0.857	0.886	0.119
	Linear growth	494.734	10	0.953	0.968	0.069
Social activity	No growth	733.015	12	0.944	0.955	0.077
	Linear growth	155.322	10	0.987	0.991	0.038
Cognitive function	No growth	1043.296	12	0.950	0.960	0.092
	Linear growth	279.220	10	0.984	0.990	0.051

### Verification of the Mediation Effects by a Multivariate LGM

#### Fitness of the Mediation Model

The chi-square value for the research model was 611.208 (*df* = 87), and the alternative hypothesis was rejected. However, the TLI and CFI were 0.984 and 0.993, and the RMSEA was 0.024 (LO = 0.022, HI = 0.026). Based on these indexes, the fitness of the research model was acceptable (Figure 1).

#### Direct Effect Between Variables

Figure 1 shows pathways for direct effects between intercepts and slopes of latent factors. The direct effects are indicated in Table 4. First, the intercept of social activity had a negative impact on the intercept of physical function decline (β = −0.401, *t* = −18.193, *p* < 0.001). The rate of change of social activity had a negative impact on the rate of change of physical function decline (β = −0.397, *t* = −10.827, *p* < 0.001). This indicates that social activity was negatively related to physical function decline in baseline and greater increase in social activity was related to a greater decrease in physical function decline over time.

**TABLE 4:** Path coefficients of multivariate latent growth modeling.

**Path**	**β**	**B**	**S.E.**	**C.R.**
SA intercept → PF intercept	–0.401	–0.263	0.022	−18.193***
SA intercept → PF slope	0.034	–0.093	0.007	−4.717***
SA slope → PF slope	–0.397	–0.219	0.037	−10.827***
SA intercept → CF intercept	0.077	0.055	0.017	4.466***
SA intercept → CF slope	0.076	0.268	0.006	13.661***
SA slope → CF slope	0.524	0.375	0.027	19.403***
PF intercept → CF intercept	–0.407	–0.446	0.015	−26.658***
PF intercept → CF slope	0.046	0.249	0.005	10.151***
PF slope → CF slope	–0.352	–0.457	0.018	−19.618***
Age intercept → CF intercept	–0.201	–0.556	0.005	−44.487***
Age intercept → CF slope	0.018	0.239	0.001	15.100***
Education intercept → CF intercept	1.125	0.134	0.108	10.428***
Education intercept → CF slope	–0.002	–0.001	0.028	–0.057
Sex intercept → CF intercept	–1.462	–0.182	0.072	−20.174***
Sex intercept → CF slope	0.207	0.126	0.019	10.792***

***p < 0.001; PF, physical dysfunction; SA, social activity; CF, cognitive function.

Second, the intercept of social activity had a positive impact on the intercept of cognitive function (β = 0.077, *t* = 4.466, *p* < 0.001). The rate of change of social activity positively impacted the rate of change of cognitive function (β = 0.524, *t* = 19.403, *p* < 0.001). This indicates that social activity was positively associated with cognitive function in baseline and a greater increase in social activity was related to a greater increase in cognitive function.

Third, the intercept of physical function decline had a negative impact on the intercept of cognitive function (β = −0.407, *t* = −0.26.658, *p* < 0.001). The rate of change of physical function decline negatively influenced the rate of change of cognitive function (β = −0.352, *t* = −19.618, *p* < 0.001). This indicates that physical function decline was negatively related to cognitive function in baseline and greater increase in physical function decline was associated with a greater decrease in cognitive function.

Finally, the intercept of age had a negative impact on the intercept of cognitive function (β = −0.201, *t* = −44.487, *p* < 0.001). The intercept of age positively impacted the rate of change of cognitive function (β = 0.018, *t* = 15.100, *p* < 0.001). This indicates that higher age in the first wave was related to a greater decrease in cognitive function over time. In addition, the intercept of education level had a positive impact on the intercept of cognitive function (β = 1.125, *t* = 10.428, *p* < 0.001). The intercept of education level had no effect on the rate of change of cognitive function (β = −0.002, *t* = −0.057, *p* > 0.05). This indicates that a higher level of education was positively associated with cognitive function in the first wave. The intercept of sex had a negative influence on the intercept of cognitive function (β = −1.462, *t* = −20.174, *p* < 0.001) and positively affected the rate of change of cognitive function (β = 0.207, *t* = 10.792, *p* < 0.001). The results indicate that cognitive function was lower in female participants than male ones.

#### Indirect Effect of Physical Function Decline

A bias-corrected bootstrap test was performed to examine the mediating effect of physical function decline on the association between social activity and cognitive function. The indirect effect of the initial value of social activity had a positive impact on the initial value of cognitive function through the initial value of physical function decline (β = 0.117, *p* < 0.01, CI = LO.105, UP.135). Also, the direct effect of the initial value of social activity influenced the initial value of cognitive function (β = 0.077, *t* = 4.466, *p* < 0.001). These results indicated that the initial value of physical function decline had a partial mediating effect between initial value of social activity and cognitive function.

Additionally, the indirect effect of the rate of change of social activity positively affected the rate of change of cognitive function through the rate of change of physical function decline (β = 0.100, *p* < 0.01, CI = LO.081, UP.119). The rate of change of social activity had a direct effect on the rate of cognitive function (β = 0.524, *t* = 19.404, *p* < 0.001). The results indicated that the rate of change of physical function decline had a partial mediating impact between the rate of change of social activity and cognitive function.

### Change Trends in Cognitive Function by Level of Social Activity

**FIGURE 2:**
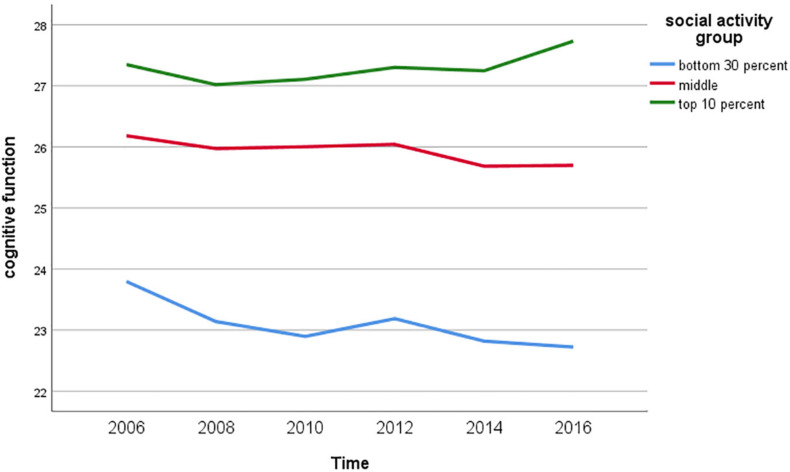
Cognitive function according to the level of social activity.

Figure 2 shows the change trends in cognitive function by the level of social activity. Cognitive function in the group with low social activity (bottom 30 percent) tended to decrease over time, while it decreased slightly in the group with moderate social activity and showed a slight increase over time in the group with high social activity (top 10 percent).

**FIGURE 3:**
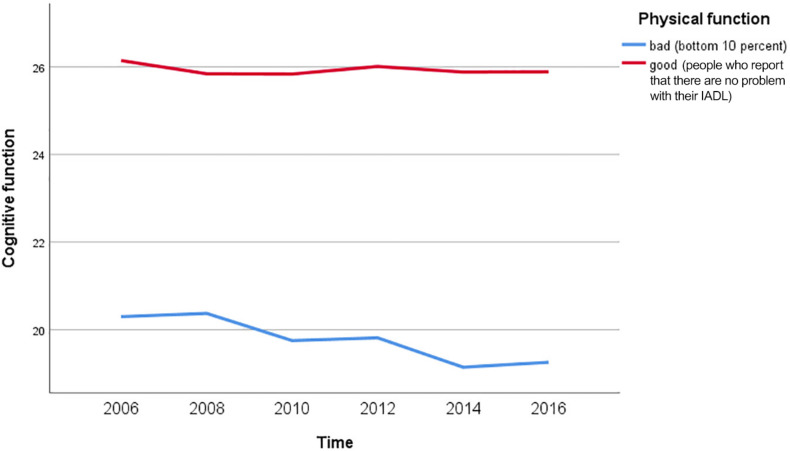
Cognitive function according to the level of physical function decline.

Figure 3 indicate the change trends in cognitive function by the level of physical function decline. Cognitive function in the group with bad physical function (bottom 10 percent) decreased to the level of mild dementia over time. However, cognitive function in the group with good physical function (people who report that there are no problem with their IADL) was maintained in the normal range.

## Discussion

The purpose of this study was to examine the mediating effect of physical function decline on the association between social activity and cognitive function. The main result of this study was that social activities in middle-aged and elderly people had a positive effect on cognitive function over time. In addition, this study determined that social activity indirectly affected cognitive function by alleviating physical function decline. A discussion of the results, along with several suggestions for this study, is as follows.

First, this study revealed that an increase in social activity was associated with an increase in cognitive function. Recently, a cross-sectional study by Fu et al. (2018) among the elderly in China verified the effect of social activity on cognitive function, while adjusting for control variables such as age, smoking, drinking, hypertension, diabetes, and depression. In addition, a longitudinal study by Choi et al. (2016) indicated that a change in social activity impacted cognitive function in middle and older Korean adults. In particular, the results outlined in Figure 2 indicate a clear association between social activity and cognitive decline, and hence may serve to maintain or even improve cognitive function. Various social activities such as social gatherings, cultural activities, and volunteering can promote such cognitive activities as forming perceptions, reasoning, considering, evaluating, and contemplating that can contribute to the maintenance of cognitive function.

A number of studies did not measure various types of social activity(Lee and Kim, 2016). Our study, however, examined the long-term effect of social activity on cognitive function by measuring various types of social activities. In addition, the majority of previous research on this was conducted for western populations (Zunzunegui et al., 2003); this study, however, indicates which results can be generalized to a non-western sample.

Second, the present study found that social activity negatively affected physical function decline. Specifically, social activities can promote the performance of basic and IADL, such as community mobility, dressing, driving, and health management, which in turn can contribute to maintaining physical functioning. Most past cross-sectional studies have argued that social activities can prevent decline in physical function. By analyzing data over 10 years, the present study confirmed that social activity could alleviate physical function decline.

Third, the present study revealed physical function decline had a negative effect on cognitive function. This result is in line with those reported by past studies, which suggested greater physical function can buffer cognitive impairment (Grande et al., 2014; Vancampfort et al., 2017, 2018). Changes in physical and cognitive function are common among older individuals, and the causal mechanism between the two variables is debated widely. Specifically, a meta-analysis that utilized longitudinal data indicated that physical functioning assessed using objective measures, such as walking speed, grip strength, and chair rise time was strongly associated with cognitive function (Clouston et al., 2013).

Fourth, social activity indirectly affected cognitive function through alleviating physical function decline. This study verified the mechanism that social activity affects cognitive function. That is, social activity increase opportunity improving physical function such as activities of daily lives, which can lead to increase in cognitive function. This study provides information useful for planning strategies to maintain cognitive function in old age by identifying the longitudinal effects of social activity and physical function on cognitive function.

The contribution of this study is to verify the longitudinal effect of social activity on cognitive function through an analysis of national sample data of middle-aged adults and the elderly. It also revealed the mechanism whereby social activities affect cognitive function. The results of this study hold implications for clinical intervention. First, our findings suggest that it is necessary to establish and implement policies to support a variety of social activities as preventive efforts to reduce the social burden due to treatment of cognitive impairment (or dementia) (Kuiper et al., 2016; Aw et al., 2017). In addition, individual efforts to preserve physical function is needed to maintain cognitive function throughout advanced aging. The limitations of this study and suggestions for future analysis are as follows. This study included control variables such as age and education, but other variables such as alcohol abuse, high blood pressure, obesity, and smoking may be associated with cognitive decline. Therefore, in future studies, researchers should consider these variables to retest the fitness of the research model in this study. This study did not verify the effects of specific types of social activities on cognitive function. However, past studies suggest that the influence of social activity on cognitive function may vary depending on the type of social activity. Future studies are needed to verify this in detail.

## Data Availability Statement

Publicly available datasets were analyzed in this study. This data can be found here: “T” the Korean Longitudinal Study of Aging (KLoSA), conducted by the Korea Employment Information Service, available at https://survey.keis.or.kr/klosa/klosa01.jsp.

## Ethics Statement

Ethical approval was not provided for this study on human participants because since this study used data freely available to the public, it did not require ethical approval. The patients/participants provided their written informed consent to participate in this study.

## Author Contributions

SB performed the study design, data analysis, and writing.

## Conflict of Interest

The author declares that the research was conducted in the absence of any commercial or financial relationships that could be construed as a potential conflict of interest.

## Abbreviations

IADL: Instrumental activities of daily living
KLoSA: The Korean Longitudinal Study of Aging.

## References

[B1] Aw S. Koh G. Oh Y. (2017). Explaining the continuum of social participation among older adults in Singapore: from ‘closed doors’ to active ageing in multi-ethnic community settings. *J. Aging Stud.* 42 46–55. 10.1016/j.jaging.2017.07.002 28918821

[B2] Bae S. Lee S. Lee S. Jung S. Makino K. Harada K. (2019). The effect of a multicomponent intervention to promote community activity on cognitive function in older adults with mild cognitive impairment: a randomized controlled trial. *Complement. Ther. Med.* 42 164–169. 10.1016/j.ctim.2018.11.011 30670238

[B3] Bidzan L. Bidzan M. Pachalska M. (2016). The effects of intellectual, physical, and social activity on further prognosis in mild cognitive impairment. *Med. Sci. Monit.* 22 2551–2560. 10.12659/msm.899004 27434501 PMC4962755

[B4] Bidzan-Bluma I. Lipowska M. (2018). Physical activity and cognitive functioning of children: a systematic review. *Int. J. Environ. Res. Public Health* 15:800. 10.3390/ijerph15040800 29671803 PMC5923842

[B5] Chen T. Chang H. (2016). Developmental patterns of cognitive function and associated factors among the elderly in Taiwan. *Sci. Rep.* 6:33486. 10.1038/srep33486 PMC5025838 27633756

[B6] Choi Y. Park S. Cho K. (2016). A change in social activity affect cognitive function in middle-aged and older Koreans: analysis of a Korean longitudinal study on aging (2006-2012). *Int. J. Geriatr. Psychiatry* 31 912–919. 10.1002/gps.4408 26833847

[B7] Clouston S. Brewster P. Kuh D. Richards M. Cooper R. Hardy R. (2013). The dynamic relationship between physical function and cognition in longitudinal aging cohorts. *Epidemiol. Rev.* 35 33–50. 10.1093/epirev/mxs004 23349427 PMC3578448

[B8] Dugan S. Gabriel K. Lange-Maia B. Karvonen-Gutierrez C. (2018). Physical activity and physical function: moving and aging. *Obstet. Gynecol. Clin. North Am.* 45 723–736. 30401553 10.1016/j.ogc.2018.07.009 PMC6226270

[B9] Frith E. Loprinzi P. (2017). The association between physical activity and cognitive function with considerations by social risk status. *Eur. J. Psychol.* 13 767–775. 10.5964/ejop.v13i4.1471 29358987 PMC5763462

[B10] Fu C. Li Z. Mao Z. (2018). Association between social activities and cognitive function among the elderly in china: a cross-sectional study. *Int. J. Environ. Res. Public Health* 15:231. 10.3390/ijerph15020231 29385773 PMC5858300

[B11] Fujihara S. Tsuji T. Miyaguni Y. Aida J. Saito M. Koyama S. (2018). Does Community-level social capital predict decline in instrumental activities of daily living? A JAGES prospective cohort study. *Int. J. Environ. Res. Public Health* 16:828. 10.3390/ijerph16050828 30866468 PMC6427449

[B12] Glei D. Landau D. Goldman N. Chuang Y.-L. Rodríguez G. Weinstein M. (2005). Participating in social activities helps preserve cognitive function: an analysis of a longitudinal, population-based study of the elderly. *Int. J. Epidemiol.* 34 864–871. 10.1093/ije/dyi049 15764689

[B13] Grande G. Vanacore N. Maggiore L. Cucumo V. Ghiretti R. Galimberti D. (2014). Physical activity reduces the risk of dementia in mild cognitive impairment subjects: a cohort study. *J. Alzheimers Dis.* 39 833–839. 10.3233/jad-131808 24296815

[B14] Hong S. (2000). The criteria for selecting appropriate fit indices in structural equation modeling and their rationales. *Korea. J. Clin. Psychol.* 19 161–177.

[B15] Hsu H. C. (2007). Does social participation by the elderly reduce mortality and cognitive impairment? *Aging Ment. Health* 11 699–707. 10.1080/13607860701366335 18074257

[B16] Hughes T. Flatt J. Fu B. Chou C. Chang H. Gangu M. (2013). Engagement in social activities and progression from mild to severe cognitive impairment: the MYHAT study. *Int. Psychogeriatr.* 25 587–595. 10.1017/s1041610212002086 23257280 PMC3578022

[B17] James B. Boyle P. Buchman A. David B. (2011). Relation of late-life social activity with incident disability among community-dwelling older adults. *J. Gerontol. A Biol. Sci. Med. Sci.* 66 467–473. 10.1093/gerona/glq231 21300745 PMC3055280

[B18] Kang Y. Na D. Han S. (1997). A Validity study on the Korean Mini-Mental State Examination (K-MMSE) in dementia patients. *J. Korea. Neurol. Assoc.* 15 300–308.

[B19] Kim D. (2016). Correlation between physical function, cognitive function, and health-related quality of life in elderly persons. *J. Phys. Ther. Sci.* 28 1844–1848. 10.1589/jpts.28.1844 27390430 PMC4932071

[B20] Kuiper J. Zuidersma M. Zuidema S. (2016). Social relationships and cognitive decline: a systematic review and meta-analysis of longitudinal cohort studies. *Int. J. Epidemiol.* 45 1169–1206. 27272181 10.1093/ije/dyw089

[B21] Lawton M. Brody E. (1969). Assessment of older people: self-maintaining and instrumental activities of daily living. *Gerontologist* 9 179–186. 10.1093/geront/9.3_part_1.179 5349366

[B22] Lee S. Kim Y. (2016). Which type of social activities may reduce cognitive decline in the elderly?: a longitudinal population-based study. *BMC Geriatr.* 16:165. 10.1186/s12877-016-0343-x 27677321 PMC5039914

[B23] Lin W. (2017). A study on the factors influencing the community participation of older adults in China: based on the CHARLS 2011 data set. *Health Soc. Care Commun.* 25 1160–1168. 10.1111/hsc.12415 28178751

[B24] Shin J. Ahn J. Kim K. (2016). Aging research panel (KLoSA) basic analysis report. *Korea. Employm. Inform. Serv.* 38 949–960.

[B25] Su X. Huang X. Jin Y. Wan S. Han Z. (2018). The relationship of individual social activity and cognitive function of community chinese elderly: a cross-sectional study. *Neuropsychiatr. Dis. Treat.* 14:2149. 10.2147/ndt.s160036 30197518 PMC6113942

[B26] Tomioka K. Kurumatani N. Hosoi H. (2016). Association between social participation and instrumental activities of daily living among community-dwelling older adults. *J. Epidemiol.* 26 553–561. 10.2188/jea.je20150253 27180933 PMC5037253

[B27] Tomioka K. Kurumatani N. Hosoi H. (2017). Association between social participation and 3-year change in instrumental activities of daily living in community-dwelling elderly adults. *J. Am. Geriatr. Soc.* 65 107–113. 10.1111/jgs.14447 27673582

[B28] Tomioka K. Kurumatani N. Saeki K. (2018). The differential effects of type and frequency of social participation on IADL declines of older people. *PLoS One* 13:e0207426. 10.1371/journal.pone.0207426 30462711 PMC6248949

[B29] Vancampfort D. Lara E. Stubbs B. Swinnen N. Probst M. Koyanagi A. (2018). Physical activity correlates in people with mild cognitive impairment: findings from six low-and middle-income countries. *Public Health* 156 15–25. 10.1016/j.puhe.2017.12.002 29408186

[B30] Vancampfort D. Stubbs B. Lara E. Vandenbulcke M. Swinnen N. Koyanagi A. (2017). Mild cognitive impairment and physical activity in the general population: Findings from six low and middle-income countries. *Exp. Gerontol.* 100 100–105. 10.1016/j.exger.2017.10.028 29104092

[B31] Xiang X. An R. (2015). Body weight status and onset of cognitive impairment among US middle-aged and older adults. *Arch. Gerontol. Geriatr.* 60 394–400. 10.1016/j.archger.2015.02.008 25747849

[B32] Zunzunegui M. Alvarado B. Del Ser T. (2003). Social networks, social integration, and social engagement determine cognitive decline in community-dwelling Spanish older adults. *J. Gerontol. B Psychol. Sci. Soc. Sci.* 200 S93–S100. 10.1093/geronb/58.2.s93 PMC3833829 12646598

