# Supplementary material for: The Mediating Effect of Physical Function Decline on the Association Between Social Activity and Cognitive Function in Middle and Older Korean Adults: Analyzing Ten Years of Data Through Multivariate Latent Growth Modeling
Source: Front Psychol. 2020 Dec 18;11:2008. doi: 10.3389/fpsyg.2020.02008 (PMC7775571; doi:10.3389/fpsyg.2020.02008)
Supplement: Supplementary file 1 [file Table_1.DOCX]

Table 1. Response rate, Respondents, Mean age, and sex ratio N=10,240

| Year | Response rate | Respondents | Age | | Gender | |
| --- | --- | --- | --- | --- | --- | --- |
|  |  |  | Mean | SD | Male | Female |
| 2006 | 100 | 10240 | 61.66 | 11.061 | 4463 | 5777 |
| 2008 | 84.78 | 8681 | 63.62 | 10.876 | 3766 | 4915 |
| 2010 | 77.29 | 7915 | 65.25 | 10.521 | 3411 | 4504 |
| 2012 | 73.08 | 7484 | 66.79 | 10.203 | 3215 | 4269 |
| 2014 | 68.63 | 7028 | 68.29 | 9.901 | 2987 | 4041 |
| 2016 | 64.62 | 6617 | 70.80 | 9.597 | 2781 | 3836 |

Table 2. Descriptive statistics of the variables

|  | Min | Max | Mean | S.D | N |
| --- | --- | --- | --- | --- | --- |
| PF1(2006) | 7 | 50 | 11.63 | 5.801 | 10240 |
| PF2(2008) | 10 | 50 | 11.66 | 5.979 | 8681 |
| PF3(2010) | 10 | 50 | 11.84 | 6.602 | 7915 |
| PF4(2012) | 10 | 50 | 11.79 | 6.448 | 7483 |
| PF5(2014) | 10 | 50 | 11.92 | 6.671 | 7028 |
| PF6(2016) | 10 | 50 | 11.99 | 6.654 | 6617 |
| SA1(2006) | 0 | 32 | 4.30 | 4.463 | 10240 |
| SA2(2008) | 0 | 27 | 4.22 | 4.018 | 8681 |
| SA3(2010) | 0 | 28 | 3.82 | 3.748 | 7915 |
| SA4(2012) | 0 | 37 | 3.87 | 3.750 | 7483 |
| SA5(2014) | 0 | 31 | 3.96 | 3.646 | 7028 |
| SA6(2016) | 0 | 24 | 3.80 | 3.671 | 6617 |
| CF1(2006) | 0 | 30 | 25.43 | 5.324 | 10033 |
| CF2(2008) | 0 | 30 | 25.20 | 5.303 | 8370 |
| CF3(2010) | 0 | 30 | 25.12 | 5.514 | 7484 |
| CF4(2012) | 0 | 30 | 25.30 | 5.516 | 7112 |
| CF5(2014) | 0 | 30 | 25.09 | 5.605 | 6657 |
| CF6(2016) | 0 | 30 | 25.09 | 5.521 | 6278 |

FF=Physical dysfunction, SA=Social activity, CF=Cognitive function

Table 3. Comparisons of fitted growth curve models for the variables

| Variable | Model | χ^2^(df) | df | TLI | CFI | RMSEA |
| --- | --- | --- | --- | --- | --- | --- |
| Physical dysfunction | No growth | 1764.505 | 12 | .857 | .886 | .119 |
|  | Linear growth | 494.734 | 10 | .953 | .968 | .069 |
| Social activity | No growth | 733.015 | 12 | .944 | .955 | .077 |
|  | Linear growth | 155.322 | 10 | .987 | .991 | .038 |
| Cognitive function | No growth | 1043.296 | 12 | .950 | .960 | .092 |
|  | Linear growth | 279.220 | 10 | .984 | .990 | .051 |

Table 4. Path coefficients of Multivariate Latent Growth Modeling

| Path | *β* | B | S.E. | C.R. |
| --- | --- | --- | --- | --- |
| SA intercept → PF intercept | -.401 | -.263 | .022 | -18.182^***^ |
| SA intercept → PF slope | .034 | -.092 | .007 | -4.695^***^ |
| SA slope → PF slope | -.396 | -.217 | .037 | -10.756^***^ |
| SA intercept → CF intercept | .120 | .086 | .017 | 6.873^***^ |
| SA intercept → CF slope | .070 | .246 | .006 | 12.487^***^ |
| SA slope → CF slope | .527 | .378 | .027 | 19.951^***^ |
| PF intercept → CF intercept | -.388 | -.424 | .015 | -25.227^***^ |
| PF intercept → CF slope | .044 | .235 | .005 | 9.541^***^ |
| PF slope → CF slope | -.348 | -.454 | .018 | -19.424^***^ |
| Age intercept → CF intercept | -.196 | -.540 | .005 | -43.113^***^ |
| Age intercept → CF slope | .017 | .235 | .001 | 14.814^***^ |
| Education intercept → CF intercept | 1.531 | .184 | .105 | 14.626^***^ |
| Education intercept → CF slope | -.041 | -.024 | .027 | -1.535 |

^*^*p*<.05, ^***^*p*<.001; PF= physical dysfunction, SA=social activity, CF=cognitive function
